# Mother, child and adolescent health outcomes in two long-term refugee camp settings at the Thai-Myanmar border 2000–2018: a retrospective analysis

**DOI:** 10.1017/S146342362400015X

**Published:** 2024-05-09

**Authors:** Marie T. Benner, Oliver Mohr, Wiphan Kaloy, Ammarat Sansoenboon, Aree Moungsookjarean, Peter Kaiser, Verena I. Carrara, Rose McGready

**Affiliations:** 1 Independent Researcher, Streithausen, Germany; 2 Independent Researcher, Bad Krozingen, Germany; 3 Malteser International, Mae Sariang, Thailand; 4 National Professional Officer (Border Migrant Health and EPI), World Health Organization, Ministry of Public Health, Nonthaburi, Thailand; 5 Mental Health and Medical Director, Swiss Red Cross Center for Victims of Torture and War, Wabern, Germany; 6 Shoklo Malaria Research Unit, Mahidol-Oxford Tropical Medicine Research Unit, Faculty of Tropical Medicine, Mahidol University, Mae Sot, Thailand; 7 Centre for Tropical Medicine and Global Health, Nuffield Department of Medicine, University of Oxford, Oxford, UK; 8 Institute of Global Health, Faculty of Medicine, University of Geneva, Geneva, Switzerland

**Keywords:** adolescent, child, maternal, primary health care, refugees, Southeast Asia, sustainable development goals

## Abstract

**Aim:**

The study assessed mothers, children and adolescents’ health (MCAH) outcomes in the context of a Primary Health Care (PHC) project and associated costs in two protracted long-term refugee camps, along the Thai-Myanmar border.

**Background:**

Myanmar refugees settled in Thailand nearly 40 years ago, in a string of camps along the border, where they fully depend on external support for health and social services. Between 2000 and 2018, a single international NGO has been implementing an integrated PHC project.

**Methods:**

This retrospective study looked at the trends of MCAH indicators of mortality and morbidity and compared them to the sustainable development goals (SDGs) indicators. A review of programme documents explored and triangulated the evolution and changing context of the PHC services, and associated project costs were analysed. To verify changes over time, interviews with 12 key informants were conducted.

**Findings:**

While maternal mortality (SDG3.1) remained high at 126.5/100,000 live births, child mortality (SDG 3.2) and infectious diseases in children under 5 (SDG 3.3) fell by 69% and by up to 92%, respectively. Maternal anaemia decreased by 30%; and more than 90% of pregnant women attended four or more antenatal care visits, whereas 80% delivered by a skilled birth attendant; caesarean section rates rose but remained low at an average of 3.7%; the adolescent (15–19 years) birth rate peaked at 188 per 1000 in 2015 but declined to 89/1000 in 2018 (SDG 3.7).

**Conclusion:**

Comprehensive PHC delivery, with improved health provider competence in MCAH care, together with secured funding is an appropriate strategy to bring MCAH indicators to acceptable levels. However, inequities due to confinement in camps, fragmentation of specific health services, prevent fulfilment of the 2030 SDG Agenda to ‘Leave no one behind’. Costs per birth was 115 EURO in 2018; however, MCAH expenditure requires further exploration over a longer period.

## Background and study area

For nearly 40 years, refugees from Myanmar have been fleeing to Thailand, India and Bangladesh, to seek refuge from human rights abuses by the authoritarian regime in Myanmar and from fights between armed opposition groups and the Myanmar military. On the Thai side of the border, camps for displaced people and predominantly of the Karen ethnic group were established in 1984 and long been regarded as a *forgotten crisis*. At the end of 2018, approximately 87 000 refugees from Myanmar were housed in nine official camps along the border, from Mae Hong Son province in the north to Ratchaburi province, southwest of the capital Bangkok. These refugees are fully dependent on external support for comprehensive health and social services. Several local and international Non-Government Organizations (NGOs) provide aid in close cooperation with trained camp residents to ensure access to basic services (Benner *et al.*, 2010; Benner *et al.*, 2008).

**Map 1.**
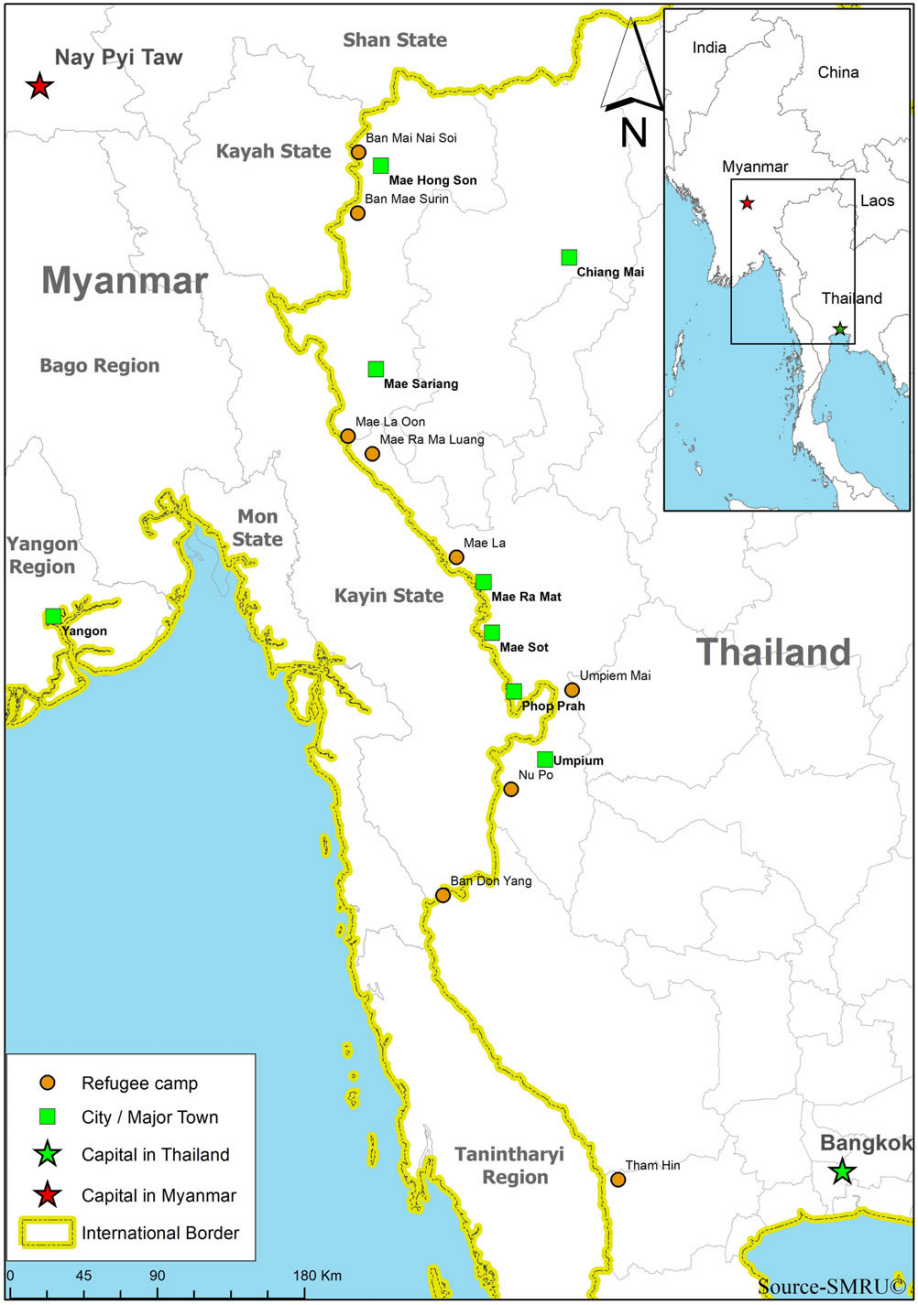
Thai-Myanmar Border Source: Myo Chit Min, Shoklo Malaria Research Unit, Mae Sot, Thailand

The study area iare two closed long-term refugee camps, Mae Ra Ma Luang (MRML) and Mae La Oon (MLO), home to 20 000 Myanmar refugees at the western border of Thailand (Map 1).

Both camps stretch over 4–5 km along rivers and through tropical forest. The nearest Thai town with a hospital is Mae Sariang which provides the closest and safest option for emergency obstetric and neonatal health services. The 80 km journey takes between 3.5 and 7 h depending on the season.

Most refugee families are now in their second or third generation. Since 2005, a substantial number of refugees have been resettled in third countries. The resettlement programme undertaken by UNHCR to date has reduced the number of refugees in Thailand by an estimated 34% (The Border Consortium, 2019). However, ‘fragility’ and sustainability regarding health remains problematic due to (i) full dependency on the international community, (ii) the continued conflict in Myanmar, (iii) endemicity of infectious diseases, (iv) mobility of these marginalized populations despite the designation of ‘closed camps’, and the (v) limitations of health services provided along the border area.

In these two isolated, and officially closed refugee camps, Malteser International (MI) has been the main INGO responsible for providing preventive, promotional and curative health care, including nutrition and Water Sanitation and Health (WASH) interventions, for 30 years (1993–2023). Birth spacing and related health education were covered by a national NGO during a limited time (2004–2013). Patients are not charged for services. MI has cooperated with the Royal Thai Government, local health authorities, national NGOs, WHO and UNHCR since 1993.

To better understand the context of long-term refugees and its potential achievements and related costs, we examined MCAH outcomes in comparison to the related SDG indicators as a benchmark, and in the context of a Primary Health Care (PHC) project and related project costs. The well-established database set by MI was used for this large analysis as well as monthly and annual reports, over a period of 18 years. Project budget reports as well as hospital-related costs were used for specific MCAH analysis. Results of infectious disease morbidity and mortality are shown in the companion article (Mohr *et al.*, 2022).

## Methods

The study includes four components: (i) a retrospective analysis of health indicators from the Health Information System database of Malteser International from 2000 to 2018, (ii); a cross-check of project reports for better interpretation of data findings from 2000 to 2018; (iii) an analysis of emergency service costs of MCAH outcomes; and (iv) interviews, carried out in January 2018, with 12 key informants; interviews were done with health and programme staff working in this setting since more than 15 years, to verify the changes of the activities over time and informing the timeline.

### Partners

Coordination with stakeholders addressing the needs of the refugees in Thailand as well as funding for these two camps has been summarized (Supplementary Material file 1). Local actors and counterparts are the Provincial and District Health Departments and hospitals as well as the Karen Refugee Committee and the Karen Women’s Organization. Patients may be referred to the district hospital in Mae Sariang or to the University hospital in Chiang Mai, Thailand for secondary and tertiary health services. A Memorandum of Agreement (MoA) of full payment for secondary care was established between MI and the hospitals.

National protocols for communicable and non-communicable diseases, Expanded Program for Immunization (EPI) and surveillance procedures for the most infectious diseases are applied, likewise for definitions of diseases including for at-risk pregnancies (Supplementary Material Table 1). Reports are directly submitted to the respective local health authorities, Bureau of Epidemiology at the Thai Ministry of Public Health who closely cooperates with the World Health Organization (WHO) and the Myanmar Health authorities, in case of outbreaks.

### Maternal, child and adolescent health

Maternal health care followed the Minimum Initial Service Package recommended by United Nation Population Fund (UNFPA) but not to the full extent according to Basic Emergency Obstetric New-born Care guideline and Comprehensive Emergency Obstetric and New-born Care (SPHERE Association, 2018), as vacuum extraction for vaginal delivery was not performed in the camps. Antenatal care (ANC) at the first visit included diagnostic for malaria, haemoglobin, urine stick, syphilis test, voluntary counselling and testing for HIV/AIDS, nutrition status and vital signs.

Cases requiring referral from the camps included all complicated deliveries, and early pregnancy complications such as ectopic, molar pregnancy and incomplete abortion. The complement of maternal, newborn and child health services are summarized (Supplementary Material Table 2). The vaccination schedule followed the Thai National EPI policy while vaccine was provided free by the Thai Ministry of Public Health and enforced by the WHO in Thailand. Birth certificates, provision of vaccines, phenylketonuria medication and hypothyroidism medication were supported by the Royal Thai Government and related Ministries (Supplementary Material Table 2).

### Data source

The MI database used was the most credible and accessible source available.

Demographic data were based on MI headcounts collected monthly through Community Health Workers (CHW) household assessments. Project reports for donors and the Thai Government were used as additional sources for the analysis on financing and programme strategies.

Target health indicators from the SDG 3 (3.1, 3.2, 3.3, 3.7, 5), SDG 6 and the refugee health programme which had 59 indicators for maternal and adolescent health were reviewed. Of those, we selected twelve key indicators (Supplementary Material Table 1): maternal mortality rate (MMR), delivery by skilled birth attendants, live birth by caesarean section, low birth weight, abortions, age-specific pregnancy rate 15–19 years, ANC visits, diphtheria/tetanus vaccination coverage, anaemia, AIDS malaria and malnutrition. For child health, 27 performance and 25 morbidity indicators were available, of which we selected twelve (Supplementary Material Table 1): Perinatal mortality rate (PNMR), neonatal mortality rate (NMR), infant mortality rate (IMR), under-five mortality rate (U5MR), global acute malnutrition (GAM,) dysentery, diarrhoea, lower respiratory infections, upper respiratory infections, malaria, skin diseases and Vitamin B1 deficiency (beriberi). The selection was based on the most relevant indicators for this context and for a long-term humanitarian setting. Relevance to other international studies with mortality data extracted from health facility bases and camp-administered records was an additional factor.

Monthly camp reports for 2000–2018, including morbidity and mortality data, were compiled into annual data sets and entered into a spreadsheet by the research team, to show annual trends. Malnutrition data were available only as proportions as the agency did not directly collect data but rather used data from external surveys. There were only a few data gaps identified over the large timeframe. These data gaps are shown in Supplementary Material Table 1. Data on birth spacing was not available.

As there were no significant differences in socio-demographic characteristics between camp populations, camp data were pooled for analysis. Host country and country-of-origin data were the comparator for maternal mortality ratio from WHO (World Data Atlas, n.d.), and the same applied for malnutrition rates (The Border Consortium *et al.*, 2017).

### Qualitative data

**Figure 1.**
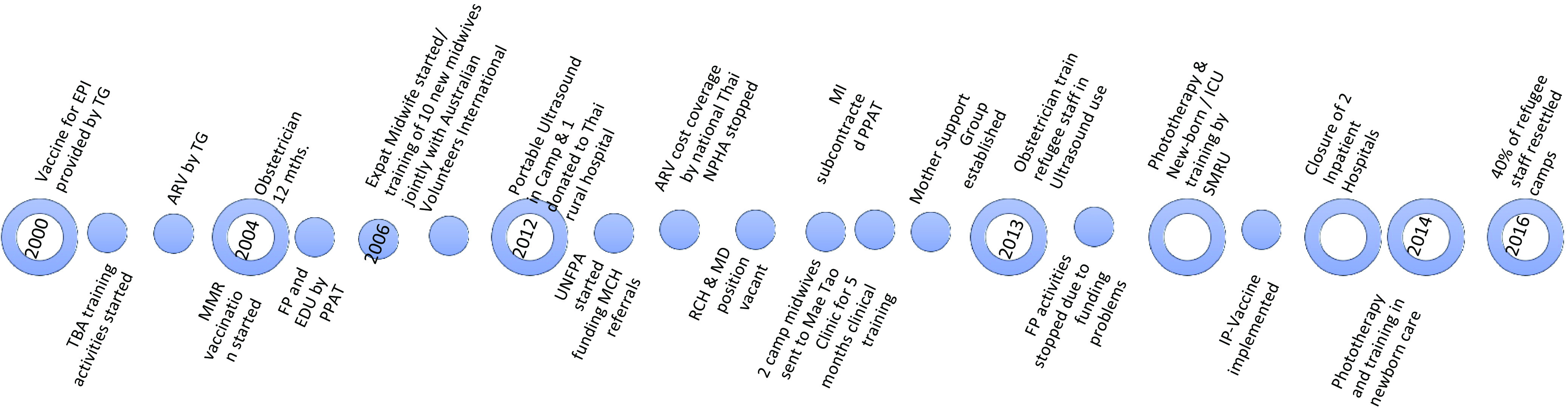
Intervention timeline

Open-ended key informant interviews (Mays and Pope, 2000) were used to capture background knowledge, diverse experiences, perspectives, to develop and interpret the timeline matrix. Furthermore, the open-ended interviews with 12 key health and WASH providers were held to explore the evolution and the changing context of the primary healthcare services over the 18 years but also to obtain an in-depth understanding of the potential impact of the resettlement programme on the health service delivery, quality and the health outcomes. Different interventions at different times and special events contributed to the timeline demonstrated in (Figure 1).

### Ethics statement

This research involves only previously collected, fully anonymized, non-government organization health data collated by the team at the Malteser International Office at 234 Moo 12, Laeng Phanit Rd, Ban Kad, Mae Sariang, Thailand, and cannot be traced back to an individual by the researcher and collaborators, and involves no biological samples; therefore, ethical approval was not required. Nevertheless, administrative permission was requested and granted by the Camps Leadership, Head of Asia Department and the Country Director of Malteser International. All authors confirm that all methods were carried out in accordance with relevant guidelines and regulations in the Declaration of Helsinki.

### Statistical analysis

Descriptive statistics were set out according to data type (continuous or categorical) and distribution (normal or skewed). The MCAH outcomes were compared to SDG health indicators while incidence rates were measured as the number of new disease-specific cases in children. The 95% confidence interval of proportions was calculated using the Wilson method (Julious, 2005).

## Results

Data on the outcome of pregnancy were available from 2002 to 2018: 93% (12,849/13,746) of deliveries occurred in the camps and only 7% (897/13,746) required emergency secondary care and were referred. There was an average of 116 obstetric, neonatal and/or infant care cases per year which required secondary emergency care alone. In 2012, almost half, 45% (126 500 EUR) of the total secondary care expenditure (230 000 EUR for 452 referrals), was incurred by 201 MCAH referrals. Pre-eclampsia, ante-partum haemorrhage and prolonged labour were the most common reason to refer; low birth weight/prematurity, pre-eclampsia and neonatal sepsis were responsible for the highest expenditures. In 2013, the planned budget for secondary care was exceeded nearly two-fold (102 713 EUR/56,380 EUR) due to the high costs related to these top three expenditures. The funding per refugee increased over the timeframe by 48% with per capita funds increasing due to the reduction in camp population (Table 1). Exchange rates as well as increases in prices for medicine and referrals influenced the budget drastically from 2017 onwards.

**Table 1.** Budget for the refugee camps MLO and MRML, 2005–2018

Year	No of total population	Budget (Euro)	Donor	Unit costs (Euros/Beneficiary/Year)
2005	27 186	740 000	ECHO	27.2
2006	30 296	909 000	ECHO	30.0
2007	31 332	950 000	ECHO	30.3
2008	33 181	1 070 000	ECHO	32,2
2009	34 492	1 200 000	ECHO, EuropeAid	34.8
2010	35 569	1 200 000	ECHO, EuropeAid	33.7 (1 EUR = 44,82 THB) 12.03.2010
2011	33 904	1 200 000	ECHO, EuropeAid	35.4
2012	32 234	1 085 000	ECHO, EuropeAid, Global Fund	33.5
2013	29 714	1 105 000	ECHO, EuropeAid, Global Fund	37.2
2014	27 085	960 000	ECHO, EuropeAid, Global Fund	35.4
2015	23 501	950 000	ECHO, EuropeAid, Global Fund	40.4 (1 EUR = 34,81 THB 17.03.2015
2016	21 802	830 000	ECHO, EuropeAid, Global Fund	38.1
2017	20 462	890 000	EuropeAid, ECHO (up to 14.02.2017)	43.5
2018	20 315	1 060 000	EuropeAid	52.1 (1 EUR = 38,40 THB) 17.03.2018

Remark: The budgets for 2016–2018 were not on a yearly base therefore we used annual estimates. The unit costs do not include patients from outside the refugee camps in the denominator (Thais, Migrants). Furthermore, the devaluation of the EURO over time has not resulted in lowering of the budget when converting into THB (https://www.xe.com/de/currencycharts/?from=EUR&to=THB&view=10Y), which has been the major currency to purchase goods in-country. Inflation rate over time was not included for costs of goods, referrals and salaries while there was a dramatic increase in the inflation rate from 2004 (2.8%) to 2011 (3.8%).

Source: https://data.worldbank.org/indicator/FP.CPI.TOTL.ZG?end=2019&locations=TH&start=1960&view=chart.

### Maternal mortality and morbidity

**Figure 2.**
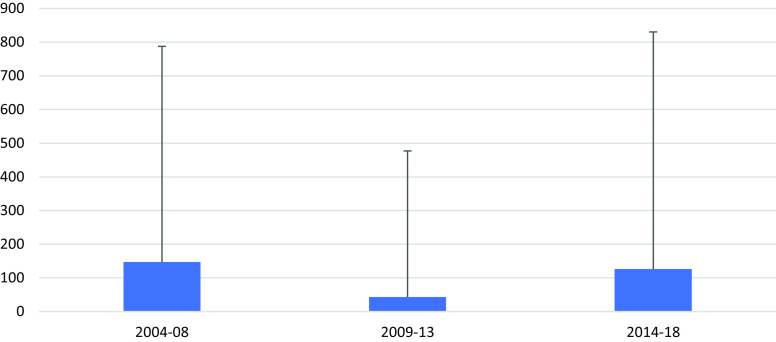
MMR per 100 000 live births (upper limit of 95% CI) in 5 years block

Ten maternal deaths relating to 12 961 live births were documented from the available data, 2004–2018 (World Health Organization, 2012). The MMR varied between 43.2/100,000 (95% CI 7.0–476.0) and 147.2/100,000 (95% CI 40.4–787.5) (Figure 2) and is not significantly higher than in Thailand (37.5/100,000) nor significantly lower than in Myanmar (247/100,000).

Four of the deaths were of unknown cause, and five of the six known causes were in grand multigravida (Supplementary Material Table 3).

### Antenatal care

**Figure 3.**
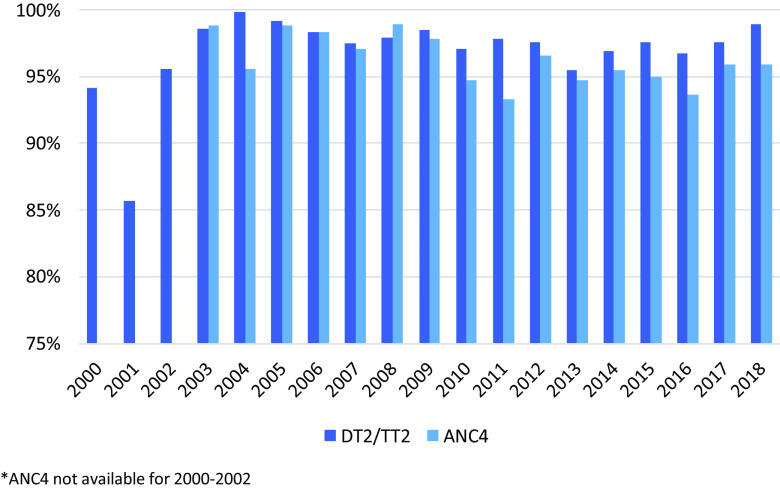
Percentage of ANC4 and DT vaccination

**Figure 4.**
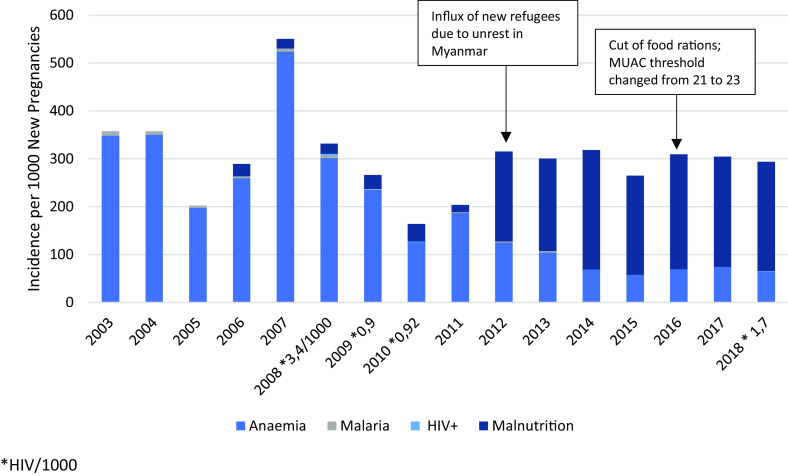
Incidence of anaemia, malnutrition, malaria, and HIV in new pregnancies at first antenatal care visit

Of the 900–1600 pregnant women per year, 93.3%–98.8% attended at least four ANC visits with >90% receiving maternal tetanus vaccine (Figure 3) throughout the years; 96.3% per year attended postnatal care. Early identification of pregnancies at risk increased from 36.2% (2009) to 61.1% (2018) of total pregnancies, partly related to an increase in youth pregnancies and grand multipara. Six cases of HIV were diagnosed between 2000 and 2018, and the proportion of women with malaria and anaemia in newly registered pregnant women at the first ANC visit declined significantly (Malaria 0/1,000; 95% CI 0.0–0.5; Anaemia 63/1,000; 95% CI 56.6–96.8); (Figure 4). The apparent increase in malnutrition goes along with a change in the definition of mid-upper-arm circumference (MUAC) malnutrition threshold from 21 to 23 (Figure 4).

### Maternal health workforce and birth outcomes

**Figure 5.**
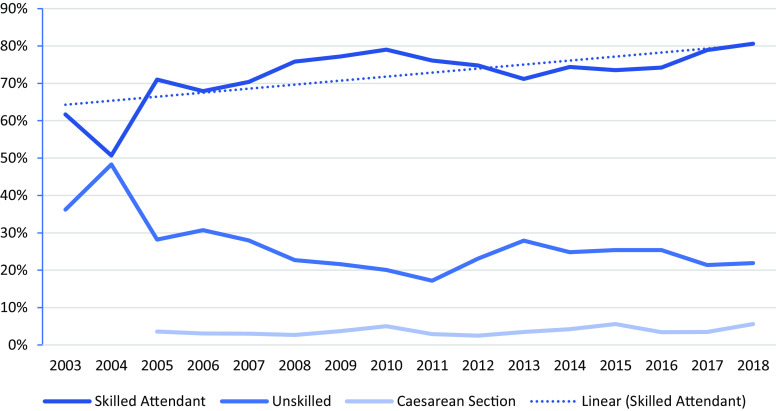
Percentage of Deliveries

Up to twenty-six midwives, 23 MCH assistants and four facilitators for the mother support groups (MSG) with an average of five years practical experience were employed in 2018 from the refugee communities, to provide antenatal and postnatal health services. The annual cost for MCAH-related expenditure, including ANC and referrals, was 60 650 EURO and about an estimate of 115 EUR per birth, in 2018. A further 110 Traditional Birth Attendants (TBAs) were active in both camps. Since 2010, midwives have been fully responsible for maternal and child health care; beforehand trained medical assistants provided these health services. Skilled and unskilled birth attendants have continued to birth women over the years, and caesarean section rates have remained extremely low, at an average of 3.6% of total deliveries (Figure 5). Deliveries by unskilled birth attendants at home were more common in MRML camp (20.3% in 2018) compared to MLO camp (14.7% in 2018), and this was similar across the study period. Stillbirth was low 187/13,590 (14/1000 live births); 1.4% (95% CI 1.2–1.6) but higher than Thailand (5/1000 live births). Low birth weight ranged from 5%–13% (2003–2017) and reflects similar results to Myanmar migrants in Thailand (Perera *et al.*, 2022) and preterm babies from 3%–6% (2004–2017) in camp-born babies.

**Figure 6.**
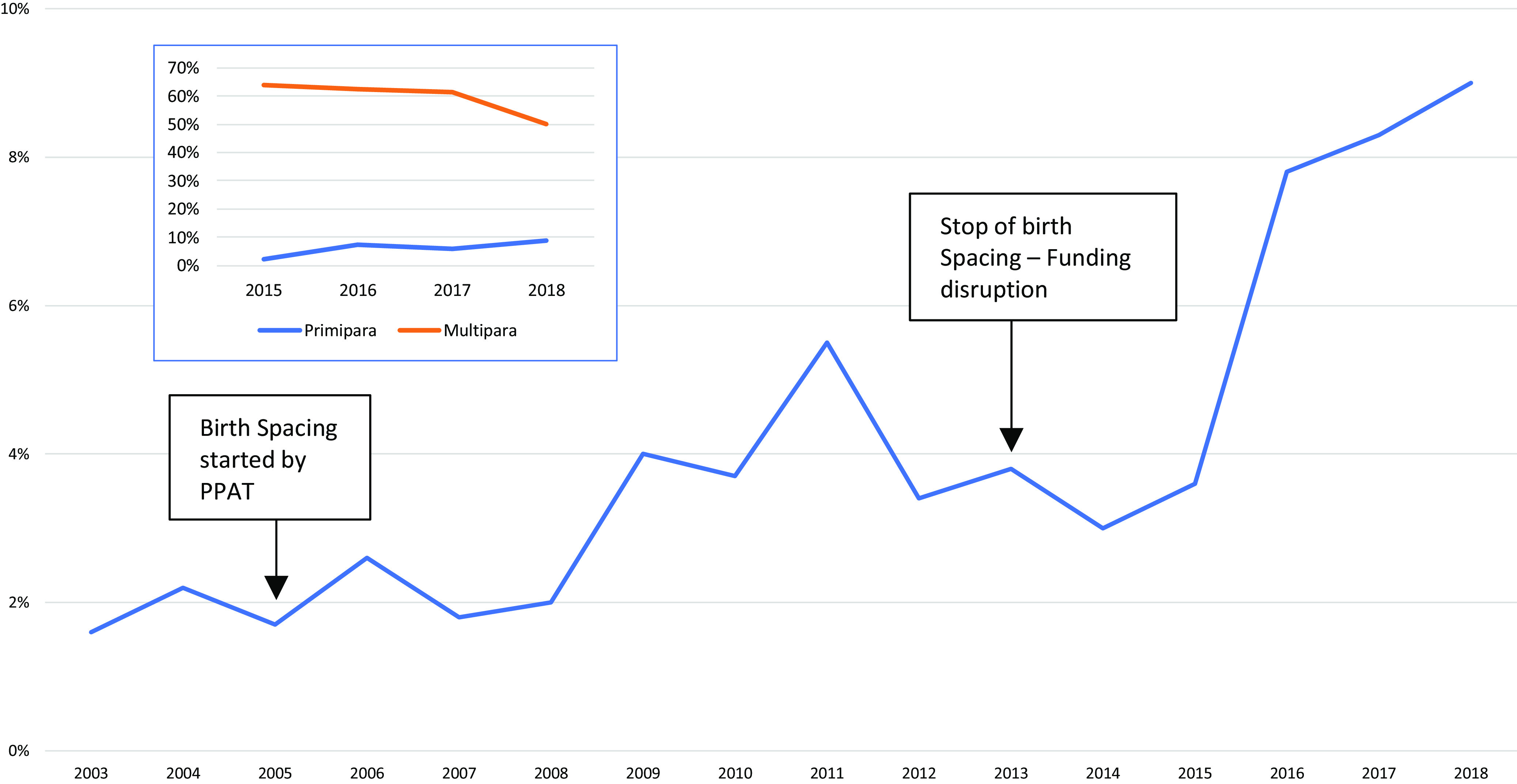
Percentage of abortions out of total deliveries (2003–2018) and by group (2015–2018)

Documented abortions have increased from 1.6% (2003) to 9% (2018) of total deliveries

(Figure 6). In 2015 and 2018, multipara women accounted for 63.8% and 50% respectively; while young women (15–19 years) accounted for 2.1% and 12.5% and the 20–24 year age group 17% and 21.4%, respectively. No maternal death related to documented abortion was recorded, and no data were available on the use of immediate post-abortion contraception.

**Figure 7.**
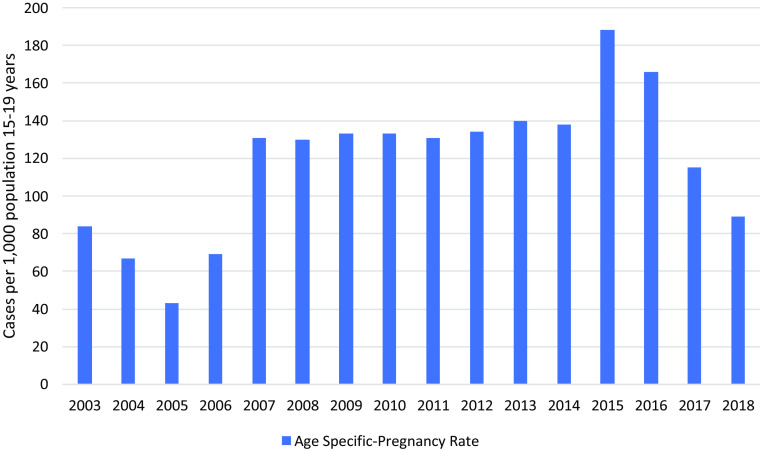
Age-specific pregnancy rate 15–19 years per thousand

The overall adolescent pregnancy rate in the two study camps was 118/1,000 women in the 15–19 life years (95% CI 99.3–137.0), with a peak in 2015 of 188/1000 (95% CI 162.0–218.0) (Figure 7). Thailand in comparison had 44.5/1,000.

### Child health

**Figure 8.**
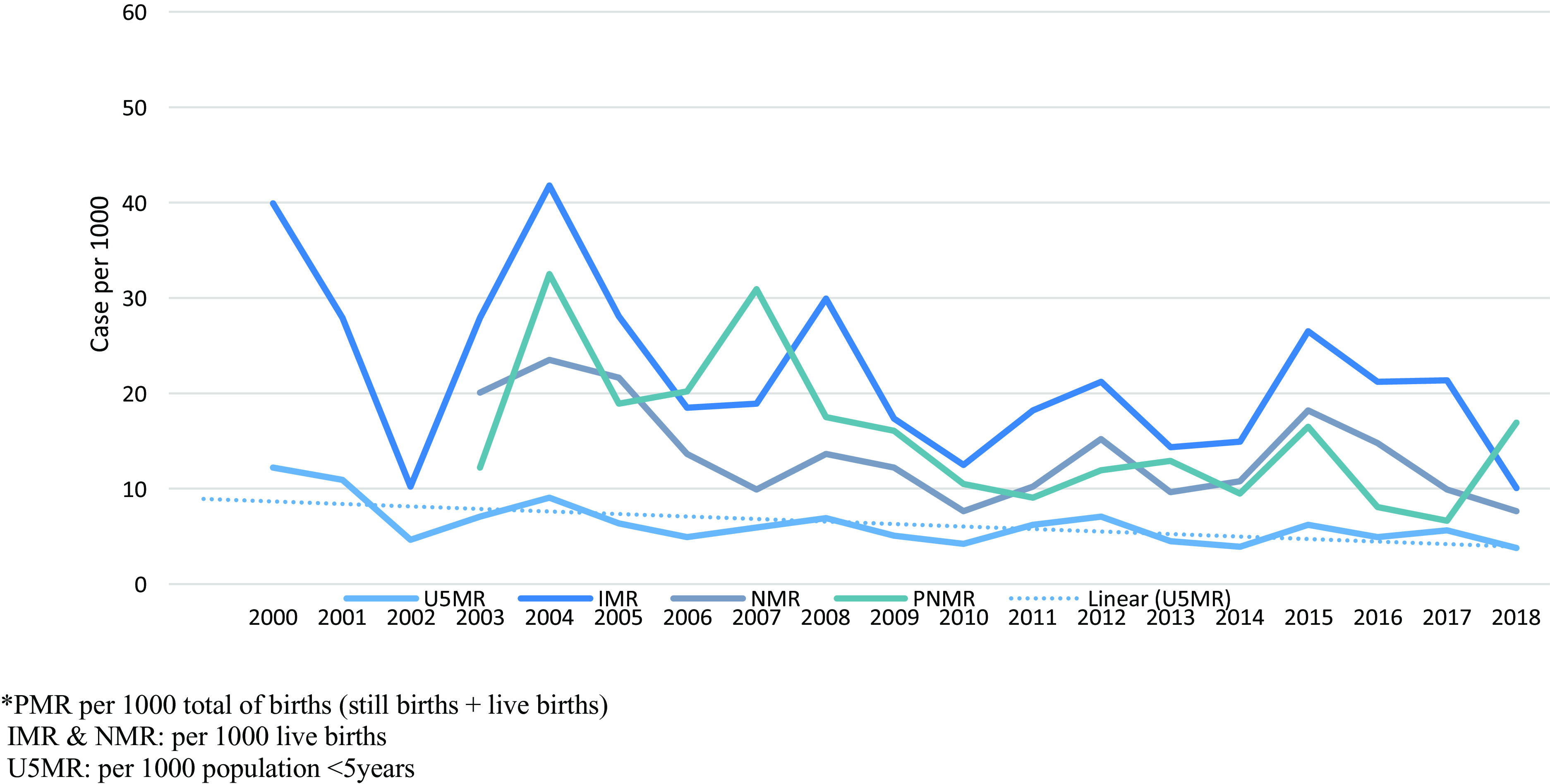
Mortality in children by age group

The proportion of children U5 has remained stable at 13%–16% of the total population, over the 18-year period. All mortality ratios decreased dramatically between 2000 and 2018: IMR from 40 (95% CI 28.0–57.0) to 10.1/1,000 (95% CI 5.5–18.4), NMR from 23.5 (95% CI 8.0–24.3) to 7.7/1,000 (95% CI 3.5–14.4) and PNMR from 32.5 (95% CI 21.5–49.6) to 16.9/1,000 (95% CI 9.9–26.0), respectively. Mortality declined by 69% in under five years old, 76% (2000–2018) in IMR, 62% (2003–2018) in NMR and 48% (2004–2018) for PNMR (Figure 8).

**Figure 9.**
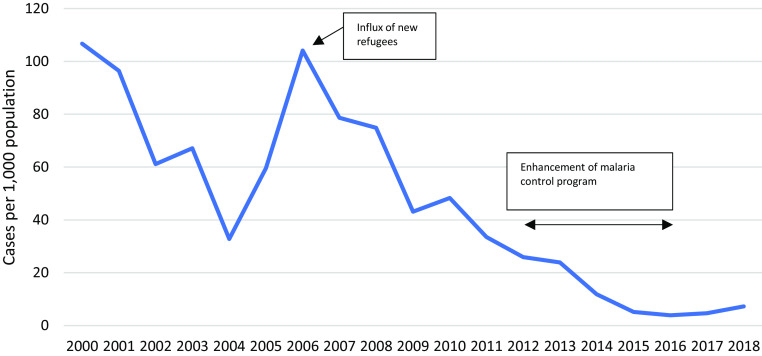
Incidence of malaria in children U5

The downward trend in incidence of malaria (all-species) in under children U5 from 2000–2018 (Figure 9) was interrupted in 2006, with a peak in incidence of over 100/1,000. During this peak, an estimated 30%–40% of overall patients came from across the border or were refugees who worked outside the camp and the overall case fatality rate increased, with a total of six deaths.

**Figure 10.**
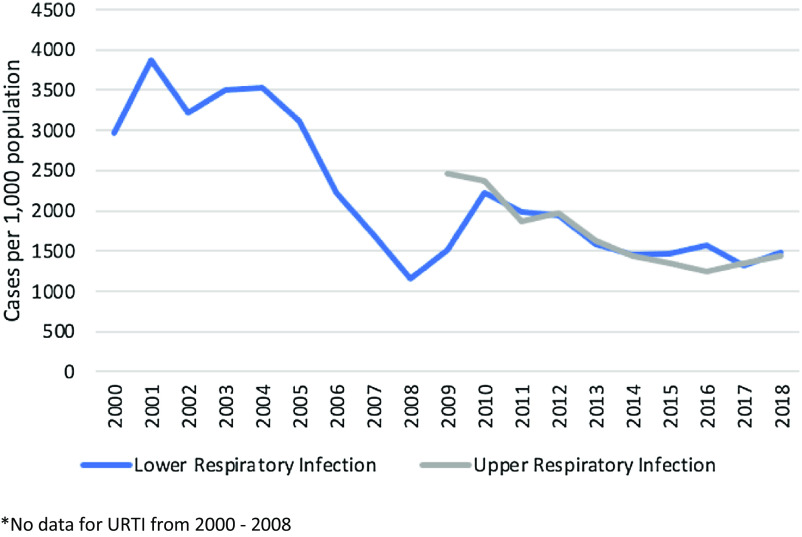
Incidence of LRTI and URTI in children U5

**Figure 11.**
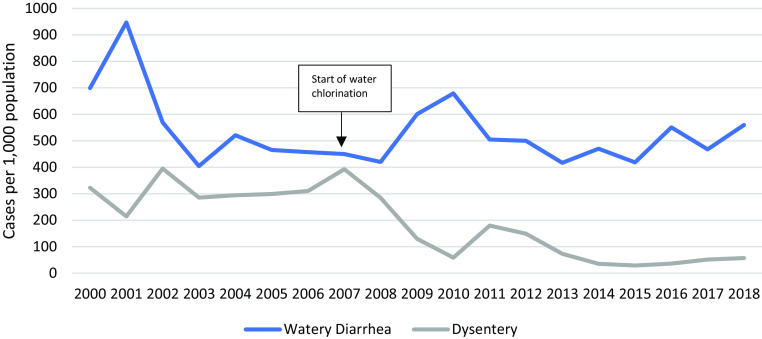
Incidence of watery diarrhoea and dysentery in children U5

**Figure 12.**
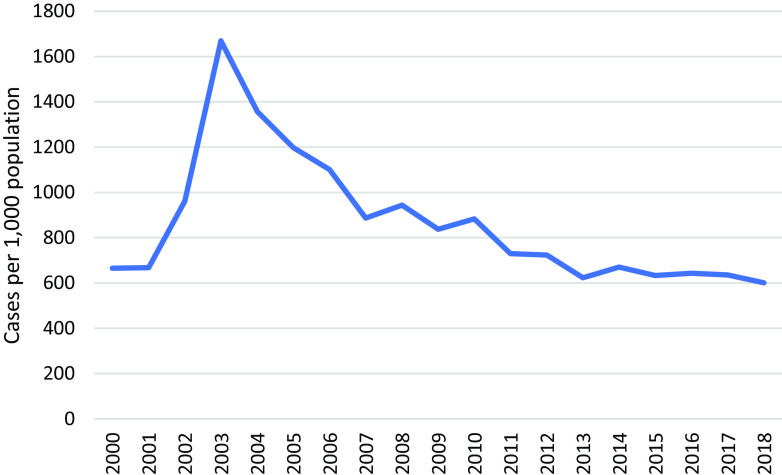
Incidence of skin disease in children U5

**Figure 13.**
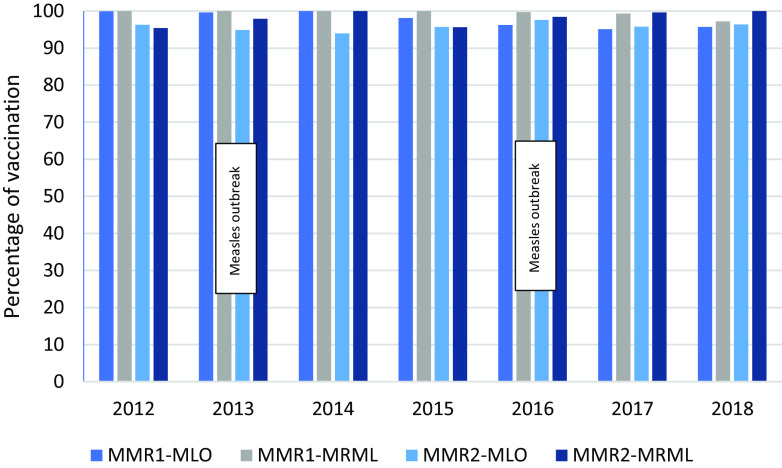
Percentage of Measles-Mumps-Rubella vaccination in children U5

Approximately half of the children had one new diarrhoea episode per year; overall respiratory tract infections, diarrhoeal disease and skin diseases in children U5 declined over the years (Figures 10–12). Measles monovalent vaccination was changed in 2012 to measles-mumps-rubella (MMR1 & MMR2) combination vaccination. Apart from MMR2 in MLO camp, vaccination rates were over 95% in both camps from 2012-2018 (Figure 13), yet, measles outbreaks were recorded in 2002, 2008, 2013 and 2015. There were no data on the 2002 outbreak, but in 2008 the first cases were imported from Eh Tu Tha, a Myanmar village with an IDP (internally displaced persons) camp, followed by an extensive measles epidemic in the surrounding villages in Myanmar. In cooperation with the local Thai district government and WHO, MI vaccinated all children between 6 months and 15 years in both camps. The 2013 outbreak in MRML camp (36 cases, incidence rate 2.4/1,000) coincided with a delayed supply of vaccine for new arrivals (HIS 2013), and the 2015 outbreak was small (12 cases and an incidence rate of 1.0/1,000), (HIS 2015).

### Nutrition

GAM in children six months to five years of age was reported at 3.9% in MRML and 4.3% in MLO camp in 2017, increasing 0.5% in MRML and 2% in MLO in 2005. These results are within acceptable levels according to the WHO classification. In 2007, the prevalence of chronic malnutrition (height for age with a cut-off <2 *z*-scores) was 38.3% in MRML camp and 41.7% in MLO camp and higher compared to the border (31.8%) and the country-of-origin Myanmar, with 35.1%, and considered as high (MRML) or very high (MLO) by WHO thresholds (de Onis *et al.*, 2019).

**Figure 14.**
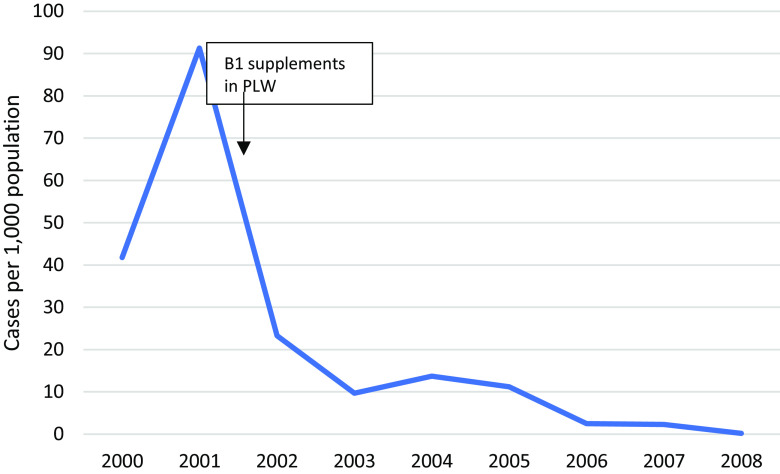
Incidence of beriberi in children U5

Bi-lateral angular stomatitis (Vitamin B2 deficiency) was highest in MLO camp at 6.9% (37/668) of the children screened and 0.4% (2/657) in MRML camp, in 2017. The incidence of infantile beriberi declined dramatically since 2002 through direct intervention with B1 supplements (Figure 14).

## Discussion

Maternal mortality did not appear to decline (126.5/100,000) despite high uptake of ANC care, decreasing anaemia and malaria and increased recognition of pregnancies at risk as well as staff development efforts and improved equipment. While detailed case studies of the deaths are not available, home birth (40%) appears problematic prior to 2014. Grand multiparity of between 6-12 previous births was undoubtedly a factor in six maternal deaths, suggesting a lack of effective birth spacing. It remains unknown whether this is an access or uptake issue. Grand multiparity is regarded as a risk in pregnancy and for neonatal complications and requires further programme adaptation. Another potential factor leading to these ten deaths is the distance to the referral hospital. Although six out of the ten mothers died in Thai hospitals, it is not known if and for how long they laboured at home. In conclusion, maternal mortality remained high and exceeded rates for Thailand (World Data Atlas, n.d.) and the SDG goal of 70/100,000 live births.

The PHC approach, implemented by one international agency, together with coordinated donor contributions, showed positive health outcomes for child mortality and morbidity. This is relevant for implementing agencies, host countries and donors alike.

Health expenditure for referrals plays an important role in adequate service provision in this setting due to the distance and harsh road conditions between the camps and the referral hospitals. We calculated an average amount of 115 EURO per birth for 2018 while more in-depth studies are needed to explore costs for MCAH. On average, 116 women and newborns annually needed lifesaving referrals to the university hospital about 5–7 h’ drive away. The caesarean section rate of 3.6% did not reach the WHO minimum threshold, which could be reflected in the high proportion of low birth weight babies (SPHERE Association, 2018).

The moderate proportion of deliveries by skilled birth attendants (Figure 5) is of concern. This has been the case for many years and increased by 19% from 2003 to 2018. Traditional birth attendants (TBAs) for ANC and more so for deliveries are promoted by a community-based organization who regard TBA activities as a major health provider if refugees are repatriated to Myanmar. TBAs are controversial and there is international discussion on the role they should take in mother and child health (WHO/UNFPA/UNICEF, 1992). An in-depth study is required to assess an association between the ANC by TBAs, home deliveries and maternal and perinatal (stillbirth and PNMR) outcomes. Intensive obstetric training support was provided during 2005 to boost the delivery skills of trained refugee midwives. Mother support groups led by TBAs learned on the importance of ANC and exclusive breastfeeding and were established in 2012. The rationale for tasking the TBAs was to use their knowledge and experience as well as their reputation within the refugee community but also to link them with the facility-based health services.

The current UNHCR-supported HIS (United Nation High Commissioner for Refugees, 2010) does not reflect the updated WHO recommendation of eight ANC visits recommended since 2016. Only deeper root cause studies would elucidate whether women understand the importance and value of the ANC/PNC services or if the additional monthly food rations (Supplementary Material Table 2) were key to the high uptake (Stuetz *et al.*, 2012).

The increase from 29.7% (2008) to 61.1% (2018) in the proportion of pregnancies identified with at least one risk criteria is most likely multifactorial and includes improved recognition by mother and child health workers; a larger proportion of primigravid (first gravida is a risk) and grand multigravida. It involves an immense workload for the midwives and the mother support groups who are aiming for positive health outcomes and prevention of complications. Interestingly, in a study in Guinea, despite a general lack of maternal health knowledge, most respondents said that ANC was important and that they would seek professional health support in case of danger signs, but they still preferred delivery by a TBA. Those exposed to health education had higher odds of facility deliveries (Howard *et al.*, 2011).

Abortion was under-recognized in early years reaching to reduced rates around 2009. Earlier research reported that herbal medicine or sticks are commonly used among women in these camps (Belton and Whittaker, 2007). Other camps along the border recorded access to misoprostol (Foster *et al.*, 2017). In the two study camps, seventeen out of 167 abortion cases were referred to secondary care during 2015–2018.

Induced abortion is prohibited in Myanmar except under life-threatening conditions (Arnott *et al.*, 2017) and until 2021it was legally restricted in Thailand, previously permitted only under certain circumstances and when mental or physical health was at risk (Arnott *et al.*, 2017). In general, abortion is a taboo subject for Karen in the camps, but informal interviews suggest refugees in the repatriation process (2016–2018), particularly pregnant women having five or more children, may have self-induced abortion as the only option, as repatriation was dependent on not being pregnant.

Both camps reported anaemia despite micronutrient supplements. Anaemia remained a chronic problem affecting 30%–40% of new pregnant women annually but improved gradually over time.

The incidence of malnutrition in new pregnant women shows a steep increase in 2012 (Figure 4), probably due to the unrest in Myanmar and a consequent large influx of new refugees (Mohr *et al.*, 2022). The MUAC threshold was raised from 21 cm to 23 cm in 2016 border-wide; however, the incidence of acute malnutrition remained constant.

MI’s policy and a strong Karen cultural traditional influence against pharmaceutical birth spacing methods reflects the limited data and practice of any form of pharmaceutical birth spacing in these camps (Benner *et al.*, 2010). Donor funding restrictions for the national implementing partner may have played an additional major role in this health outcome. This would be considered a limitation of the programme and an area for improvement with the potential to impact maternal mortality, adolescence health and reduction of abortions. Lack of political will and cultural adaptation has been a major barrier to other studies (Wardeh and Marques, 2021). Health expenditure for MCAH requires further exploration as little is known on costs for these programmes.

### Child health

A study in Mae La camp also showed a decline of 51% in NMR from 21.8 (2009) to 10.7 (2011) for infants from 28 weeks’ gestation. This was achieved by the introduction of simple low-cost unit specializing in care of sick neonates run by local refugee health workers with training support to increase staff competency (Turner *et al.*, 2013a).

In 2005, a local NGO started promotion of birth spacing and increased deliveries by skilled birth attendants during the same period (Figure 5). Birth spacing stopped again in 2013 and abortion started to rise afterwards (Figure 6). From 2003 to 2018, there were 187 stillbirths in the two study camps, but data on whether these were pre- or intrapartum were not available. Prematurity and its complications (birth asphyxia, sepsis in neonates) contributed to the deaths and are similar to the results from another border camp (Turner *et al.*, 2013a) and reported globally (UNICEF *et al.*, 2017).

Neonatal deaths in the camps started to decrease in 2015 with the introduction of phototherapy and the training of staff in special care for newborns following the Shoklo Malaria Research Unit experience described in the study by Turner (Turner *et al.*, 2013a). Newborns from the Karen ethnic group are at increased risk of neonatal hyperbilirubinemia (Bancone *et al.*, 2022; Thielemans *et al.*, 2021). Alongside this, establishing the mother support groups, increasing the number of MCH workers and six months specialist support from a paediatric doctor built local capacity.

Of the ten countries with the highest NMR (30–50/1,000), five are in Asia (India, Pakistan, China, Bangladesh, Indonesia) and the rest in Africa (Save the Children *et al.*, 2018).

In summary, the promotion and capacity building of ANC and the identification of at-risk pregnancies, safe birth services and special care baby unit are likely to have contributed to the significant decline in stillbirths and child mortality.

### Infectious diseases: malaria, respiratory tract infection, diarrhoea, measles

The early detection and prompt treatment approach with combination therapy artesunate/mefloquine for *P. falciparum* and chloroquine for non-falciparum (principally *P. viva*x) infections, introduced in 1995, has been very effective and consistent with previous publications on malaria control in the Thailand-Myanmar border camps and for migrant populations (Landier *et al.*, 2016; Carrara *et al.*, 2013; Chu *et al.*, 2020; Nosten *et al.*, 2000). It was not possible to disaggregate the HIS data on the contribution to malaria rates from *P. falciparum* and *P. vivax* although this would be helpful in the efforts to eliminate malaria in the Greater Mekong Sub region.

Global acute malnutrition in children six months to five years of age was reported at 3.9% (MRML, 2017) and 4.3% (MLO, 2017), respectively, which was higher compared to the border (2.1%) but lower than in Myanmar (7.9%) (The Border Consortium *et al.*, 2017), and ‘acceptable’ according to the WHO threshold of below 5%. Chronic malnutrition in children increased in MLO by 1.2% during the same period, relating to a new influx of arrivals from Myanmar and to food ration cuts. A strong emphasis on prevention through supplementation of vitamin B1 in antenatal clinics and during lactation, and on treatment through clear case definitions and treatment of suspected cases, has virtually eliminated morbidity and mortality from infantile beriberi (Figure 14). Up to one in four infants died of this disease in other Thai-Myanmar border camps during the nineties (Luxemburger *et al.*, 2003).

In 2008, the first measle cases were imported from Eh Tu Tha, a Myanmar village with an IDP camp, followed by an extensive measles epidemic in the surrounding villages in Myanmar. In cooperation with the local Thai district government and WHO, MI vaccinated all children between 6 months and 15 years in both camps. The 2013 outbreak in MRML camp (36 cases, incidence rate 2.4/1,000) coincided with a delayed supply of vaccine for new arrivals (HIS 2013). The 2015 outbreak was small (12 cases and an incidence rate of 1.0/1,000 (HIS 2015).

In summary, all major disease incidence rates in children U5 improved over this period. The dysentery incidence declined from 2002 onwards by 14%, upper respiratory infections by 42% (2009–2018), lower respiratory infections by 62% (2001–2018) with similar trends in neighbouring camps [29], skin diseases declined by 64% (2003–2018), vitamin B_1_ deficiency by almost 100% and malaria by 93% (2000–2018).

Tailored programmes adapted to local needs appeared to be key to the improvements in mother–child health in these two camps based in a remote setting with communication and access issues. These included a focus on infectious diseases and provision of vitamin B_1_ in the early intervention years reduced child mortality substantially, various vertical intermediate interventions such as WASH, simple improvements in technology, intensive coaching in delivery skills including competence in essential neonatal care and locally appropriate neonatal guidelines.

### Adolescents

Adolescent pregnancy rates were high and similar to rates reported 250 km further south at Mae La refugee camp, with a peak of 142/1,000 (95% CI 120.0–167.0) in 1998 (Parker *et al.*, 2018). Globally, these are high compared to 45/1000 in South-East-Asia and 7/1,000 in East Asia (UN DESA Statistic Division, 2017). Access to information, education and services related to reproductive health for non-married youth has been restricted in these camps with leaders in the area fearful of promiscuity (Benner *et al.*, 2010). However, since October 2016, young people have had access to ‘adolescent reproductive health’ information in all camp schools using a training curriculum with training of trainers (ToT) approach for teachers. Access to contraception was not available. Additionally, MSG have facilitated ‘reproductive health’ sessions for reproductive-age women since July 2015. The Royal Thai Ministry of Interior’ restriction of education beyond camp graduation, the sparsity of job opportunities, traditional cultural norms that restrict knowledge, boredom and a lack of future prospects, are all likely contributing factors (Benner *et al.*, 2010). In summary, adolescent birth rates in MLO and MRML are high and similar to Maela, the largest camp on this border. In the last three years, a successive downward trend may indicate that the specific school health curriculum is being effective.

## Limitations

This is a retrospective review in which the pooled annual mean camp population has been used as the denominator in a setting where Myanmar cross-border and Thai host populations can all access the free health services in the camp, this may have impacted the numerator. Nevertheless, as the policy to permit access did not change over the period the trends are subject to the same risk of bias throughout the period. There are other factors that may not have been considered here and may have contributed to the reduction in child morbidity and mortality.

Exclusive breastfeeding was not systematically reported (except through border-wide surveys) over the years and the border-wide HIS put more emphasis on feeding programme coverage and performance. Food security and nutrition surveys were implemented by a local NGO, while feeding programmes and growth monitoring were done by MI. Therefore, the authors were not able to assess the child nutrition data.

MI’s policy and donor financial restrictions of the national implementing partner on pharmaceutical birth spacing certainly influenced possibilities in this setting and limited the intervention in MCAH.

## Conclusion

It is well documented that people affected by conflict are more likely to die or fall ill and contribute to a certain extent to the SDGs indicators while refugee camps, in its artificial environment, were never intended to provide permanent or sustainable solutions (d’Harcourt *et al.*, 2017). Yet this is increasingly becoming the case, and these camps are becoming insolvent (Wardeh and Marques, 2021).

The political and social environment in this refugee setting, including limited education for adolescents, unemployment, migration, resettlement and fragmentation of services (nutrition, birth spacing), all contribute to chronic inequity. Nevertheless, INGOs jointly with national actors and other stakeholders can bring significant improvements and contributions towards the SDG-related health indicators through a continuous and steady improvement of health interventions related to MCAH. Sustainability of funding for services is increasingly difficult to secure with the current global increase in protracted humanitarian crises.

Of the Sustainable Development health indicators: maternal mortality (SDG3.1) could not be attained; newborn and child mortality (SDG3.2) were achieved; communicable diseases (SDG3.3) made significant progress, and sexual reproductive health services (SDG3.7) were incompletely addressed. Maternal, child and adolescent health requires attention, and the review suggests that PHC is an appropriate approach in refugee settings to address some of the health-related SDGs, but it needs to be community and patient-centred, adapted to a closed refugee setting, and context-specific to leave no one behind. Maternal mortality, adolescent health and the reduction of abortion could be improved effectively by considering a comprehensive package of services (SDG 3.7) to contribute to the sustainable development health indicators in 2030. The health information system requires adaptation, so it can be fully utilized to guide relevant needs and a border-wide programmatic strategy for protracted crises in Asia.

## Supporting information

Benner et al. supplementary material 1 Benner et al. supplementary material

Benner et al. supplementary material 2 Benner et al. supplementary material

Benner et al. supplementary material 3 Benner et al. supplementary material

## Supplementary material

To view supplementary material for this article, please visit https://doi.org/10.1017/S146342362400015X
