# Supplementary material for: Mother, child and adolescent health outcomes in two long-term refugee camp settings at the Thai-Myanmar border 2000–2018: a retrospective analysis
Source: Prim Health Care Res Dev. 2024 May 9;25:e27. doi: 10.1017/S146342362400015X (PMC11091483; doi:10.1017/S146342362400015X)
Supplement: Benner et al. supplementary material 1 — Benner et al. supplementary material [file S146342362400015Xsup001.pdf]

Supplementary Material Table 1 Indicators and Definitions

Maternal and Adolescent Indicators

Maternal Mortality Ratio (MMR) 2004-2018: number of pregnancy related deaths / total number of live births annually x 100.000 live births in a given year.

Ante Natal Care (ANC) 2003 – 2018: four visits by a pregnant woman before giving birth

Diphteria/Tetanus (dt2) and Tetanus (T2): at least two dt / tetanus per pregnant woman

Deliveries by skilled birth attendant 2003-2018: total numbers of deliveries by skilled birth attendants / total deliveries x 100 in a given year.

Low Birth Weight: any weight below 2.5kg

Proportion of live births by caesarean section (C/S) 2006-2018: number of live birth by caesarean section / number of total live births x 100 in a given year.

Age specific pregnancy rate aged 15-19 years 2003-2018: number of pregnancies women aged 15-19 years / of total 15-19 age group /1000 in a given year.

Abortion 2003-2018: Pregnancy loss of gestations <28 weeks. Abortion data by primiapara and multipara available only 2015-2018.

Anaemia at all age groups 2000-2018: Haemoglobin <10g/dl.

AIDS: positive HIV test

Malaria in pregnancy: Malaria positive with microscope in all new pregnant women

Malnutrition in pregnant women: Mid-arm-circumsference (MUAC) <21 (until 2016) /23 cm (2016-2018)

Child Indicators

Perinatal Mortality Rate (PMR) 2000-2018: death of the foetus or neonate up to 7 days of life (perinatal deaths = birth and first 7 days) / total of births (still births + live births) annually x 1000 in a given year.

Neonatal Mortality Rate (NMR) 2004-2018: deaths of neonates before reaching 28 completed days of life / total number of live births x 1000 in a given year.

Infant Mortality Rate (IMR) 2000-2018: death of infants (<1 year of age) / total number of live births annually x 1000 in a given year.

Child Mortality Rate (U5MR) 2000-2018: death of children (<5 years of age) / number of children <5 years annually x 1000 in a given year.

Lower respiratory infection incidence U5 years 2000-2018: number of new lower respiratory cases in children under 5 years / number of total populations under 5 years x 1000 in a given year.

Upper respiratory infection incidence U5 years 2000-2018: number of new upper respiratory cases in children under 5 years / number of total populations under 5 years x 1000 in a given year.

Dysentery incidence U5 years 2000-2018: number of new dysentery cases in children under 5 years / number of total populations under 5 years x 1000 in a given year.

Diarrhoea incidence U5 years 2000-2018: number of new dysentery cases in children under 5 years / number of total populations under 5 years x 1000 in a given year.

Malaria incidence U5 years 2000-2018: number of new malaria cases in children under 5 years / number of total populations under 5 years x 1000 in a given year.

Skin disease incidence U5 years 2000-2018: number of new skin disease cases in children under 5 years / number of total populations under 5 years x 1000 in a given year.

Vitamin B 1 deficiency incidence U5 years 2000-2018: number of new Vitamin B 1 deficiency cases in children under 5 years / number of total populations under 5 years x 1000 in a given year

Global Acute Malnutrition in U5 years: proprotion of children 6-59 months with Severe Acute Malnutrition (SAM) and Moderate Acute Malnutrition (MAM) measured through weight for height (WHZ) and / or oedema

Skilled birth attendant: doctors or trained midwives which included those trained in Thailand (four-year degree program) or the refugee camp staff (nine-month program following the training) curriculum of the American Refugee Committee) who can do almost all core functions such as diagnose high risk pregnancies, emergency obstetric and new born

care and carry out normal deliveries, except operative obstetrics, which requires transfer to the Thai hospital.

High risk pregnancy definition:

Maternal age <17 or >35 years	Malnutrition Mid Upper Arm Circumference (MUAC) <23cm
Grand Multigravida > 4 previous pregnancies	
**Past pregnancy history with:**	
Stillbirth or neonatal death	Prolonged labour
Baby weight <2500g or >4000g	Previous caesarean section
Premature rupture of membranes, premature onset of labour (<37 weeks)	Congenital abnormality, cerebral palsy, birth trauma, or recurrent abortion
Vaginal bleeding duirng pregnancy	Anaemia (Hb. <10gm/dL or Hct. <30%)
VDRL +ve, HIV +ve	Urine exam: Albumin +ve/Glucose +ve
Multiple pregnancy	Weight gain <1kg/month from 24 weeks
Size of the uterus not related to gestational age	Fetal movement <10times/day from 32 weeks
Pregnancy >40 weeks gestational age	Abnormal position of the fetus from 34 weeks
Medical history and/or during pregnancy BP ≥140/90 mmhg, Pregnancy induced hypertension and pre-eclampsia and eclampsia	Medical history and/or during pregnancy: Enlarged Thyroid, Diabetes, Blood disorders, Heart disease or signs during examination, chronic disease or acute maternal illness e.g. Malaria, UTI
