# Supplementary material for: Mother, child and adolescent health outcomes in two long-term refugee camp settings at the Thai-Myanmar border 2000–2018: a retrospective analysis
Source: Prim Health Care Res Dev. 2024 May 9;25:e27. doi: 10.1017/S146342362400015X (PMC11091483; doi:10.1017/S146342362400015X)
Supplement: Benner et al. supplementary material 2 — Benner et al. supplementary material [file S146342362400015Xsup002.pdf]

Supplementary Material Table 2 Maternal and new-born care services

**Antenatal Care**	Diagnostic weekly/monthly (risk/normal pregnancies): malaria, haemoglobin, urine stick, syphilis test, nutrition status (MUAC in cm & weight gain in kg) vital signs<br>Training of midwives by the RH coordinator (nurse/midwife) about complications during pregnancy and at delivery monthly.<br>Close monitoring of risk pregnant women in ANC.<br>Clean delivery kits for skilled birth attendants and traditional birth attendants<br>Anaemia: screening at first ANC visit; if no signs of anaemia a follow up check at GA 28 weeks. Pregnant women with anaemia will be rechecked after one-month treatment. Pregnant woman with Anaemia will received Ferrous Sulphate (200 mg) 1 tab oral TID and Folic acid 1 tab oral OD until her HCT reached to 30% or HB to 10g/%.<br>Supplementary food rations for pregnant women:<br>Healthy pregnant woman receive blanket supplementary feeding provides over 300 kcal/day and includes<br>• 1.5 kg Asia remix<br>• 0.5 kg pulses<br>• 0.5 L vegetable oil.<br>Micronutrient supplements included:<br>- Vitamin B1 (100 mg) 1 tab per week.<br>- Ferrous Sulphate (200 mg) 1 tab per day (Now MI changing to Ferrous Fumarate)<br>- Folic Acid (5 mg) 1 tab per week<br>Malnourished pregnant woman receive blanket supplementary feeding provides over 600 kcal/day and includes<br>• 2 kg Asia remix<br>• 0.5 kg pulses<br>• 0.5 L vegetable oil.<br>Vaccination: Diphtheria and Tetanus prophylaxis (dT)
**Postnatal Care**	Postpartum care services: daily screening of mother and baby<br>Vitamin A for mothers and babies.<br>Expanded Program for Immunization (Thai National protocol) including hepatitis b birth dose in 24 hours<br>Supplemental food rations for lactating mothers<br>Vitamin B1 (thiamine hydrochloride)
**Basic and comprehensive emergency obstetric and new-born care (incl. safe blood transfusion)**	Basic emergency obstetric and new born care partially available (parenteral antibiotics, uterotonic drugs, parenteral MgSo4 anticonvulsant, uncomplicated deliveries, manual removal of placenta, and maternal and new born resuscitation).<br>NB: uncomplicated vaginal delivery with vacuum extraction cannot be performed due to lack of vacuum equipment.

	<u>Comprehensive emergency obstetric and new born care</u> partly available, including post-abortion care, tetanus prophylaxis<br>Implementation and monitor therapeutic standards and documentation for delivery and labour<br>Phototherapy to treat treatment jaundice introduced in 2014<br>Mobile ultrasound introduced 2016
**Referral services (24h/7) for caesarean section**	Emergency car on stand-by during day time; no travel allowed at night due to security concerns; but rented car from Thai village was possible during the night time
**Health education /Behaviour Change Communication**	Promotion of exclusive breastfeeding and hygiene measures<br>Health Education through Mother Support Groups and MCH workers;<br>Promotion of deliveries with skilled birth attendants in birth facility;<br>Health Promotional Campaigns for safe motherhood, immunization, diseases prevention, nutrition
**Medical and clinical treatment of all relevant reproductive health issues and communicable and non-communicable diseases**	Diagnostic and treatment of all relevant health issues<br>Presumptive treatment of sexual transmitted infections
**HIV and AIDS /STI**	Voluntary and confidential counselling and testing (VCCT)<br>Syphilis screening<br>Provision of ARV for HIV positive pregnant women using ARV eligibility criteria<br>Safe blood transfusion<br>Precautions for health workers<br>Health prevention campaigns
**Sexual Gender Based Violence (SGBV)**	Clinical care; Post-Exposure-Prophylaxis (PEP) kit;<br>Emergency contraception; medical and referral services for SGBV survivors; psycho-social support<br>Hepatitis B vaccination<br>Community awareness (by UNHCR & MI)
**Capacity Development (regular); Empowering refugee communities**	Training in utilization of WHO partograph<br>Training in labour management<br>Training in management of eclampsia and pre-eclampsia<br>Management of postpartum haemorrhage<br>Engagement of TBA's in ANC and during deliveries<br>Engagement of mother support groups<br>Engagement MCH workers<br>Training to ensure birth registration and certification for the Thai authorities

Child health services

**Medical / Clinical Care / protection aspects**	Medical/clinical primary care services of all relevant diseases/micronutrient deficiencies for all children under 5 years; phenylketonuria medication and hypothyroidism medication<br>Prevention of Mother-to-Child transmission HIV<br>Diagnosis of major diseases<br>Referral service 12h/7<br>Birth certificate
**Post-Natal care**	Well baby clinic for all children < 1 year / monthly
**School Health**	6 monthly screening in all nurseries, primary and secondary schools<br>Hygiene education 6-monthly<br>Treatment of vitamin deficiencies<br>Deworming: Albendazole 400 mg oral stat every 6 months for children aged 2-12 years
**Growth Monitoring**	Guidance to support exclusive breastfeeding<br>Support for complementary feeding practices
**Infant and young child feeding**	Height/Age (Stunting): < 3 years of age bi-monthly; 3-5 years 6-monthly<br>Weight/Height (Wasting): 6-monthly growth monitoring of children between 36-59 months of age<br>Therapeutic feeding<br>Supplementary feeding<br>Vitamin A supplementation in children < 5 years of age (6-monthly)
**Dental Hygiene**	Daily tooth brush activities in grade 3-5<br>Dental screening unit available (5/7 days)
**Expanded Program for Immunization (EPI)**	New-borns: Hepatitis B birth dose; BCG<br>2 months: DTP-HB 1; OPV 1<br>4 months: DTP-HB 2; OPV 2; IPV<br>6 months: DTP-HB 3; OPV 3<br>9 months: MMR 1<br>12 months: JE 1<br>18 months: DTP 4; OPV 4<br>2.6 years: JE 2; MMR 2<br>4 years: DTP 5; OPV 5<br>6 years: MR (if not received MMR at 2.6 years);<br>dT, OPV (if not received DTP and OPV completed 5 doses)<br>12 years: dT<br>*Remark*: Introduction of JE, MMR vaccine in July 2013; introduction of IPV in September 2016
