# Supplementary material for: Mother, child and adolescent health outcomes in two long-term refugee camp settings at the Thai-Myanmar border 2000–2018: a retrospective analysis
Source: Prim Health Care Res Dev. 2024 May 9;25:e27. doi: 10.1017/S146342362400015X (PMC11091483; doi:10.1017/S146342362400015X)
Supplement: Benner et al. supplementary material 3 — Benner et al. supplementary material [file S146342362400015Xsup003.pdf]

Supplementary Material Table 3 Causes of Maternal Death reported between 2004 and 2018

Year	Causes of Death	ICD-10*	Place
2004	Two deaths of unknown cause	O95, O95	Camp
2005	Unknown cause	O95	Camp
9/2005 (rainy Season)	22 years, G1, with ruptured ectopic pregnancy (woman did know that she was pregnant)	O08.1	Thai Hospital
6/2010 (rainy Season)	38 years, G6 P6 D2 with unknown cause. She passed away after 2 hours after normal vaginal delivery.	O95	Thai Hospital
2013	33 years G6 P5 suspected thrombosis	O22	Camp
2015	36 years G9 P7 PPH after caesarean section and hysterectomy was done (attended camp ANC; self-referred in labor to MSR)	O72.1	Thai Hospital
2016	39 years G12 P11. APH (Abruptio placenta)	O45	Thai Hospital
2016	38 years G11 P8 A2, Heart failure (Cardiogenic shock)	O99.4	Thai Hospital
2017	36 years G8 P7 A1 PPH (Coagulopathy)	O72.1	Thai Hospital

Abbreviations G gravida, P parity, A abortions, D born alive but died

Other APH antepartum haemorrhage, MSR Mae Sariang Hospital, PPH post-partum haemorrhage,

*ICD10 [14]
